# Bone-brain interaction: mechanisms and potential intervention strategies of biomaterials

**DOI:** 10.1038/s41413-025-00404-5

**Published:** 2025-03-17

**Authors:** Jiaze Yu, Luli Ji, Yongxian Liu, Xiaogang Wang, Jing Wang, Changsheng Liu

**Affiliations:** 1 The State Key Laboratory of Bioreactor Engineering, East China University of Science and Technology, Shanghai, 200237 PR China; 2 Engineering Research Center for Biomedical Materials of the Ministry of Education, East China University of Science and Technology, Shanghai, 200237 PR China; 3 Key Laboratory for Ultrafine Materials of Ministry of Education, East China University of Science and Technology, Shanghai, 200237 PR China; 4 Frontiers Science Center for Materiobiology and Dynamic Chemistry, East China University of Science and Technology, Shanghai, 200237 PR China

**Keywords:** Bone quality and biomechanics, Neurophysiology

## Abstract

Following the discovery of bone as an endocrine organ with systemic influence, bone-brain interaction has emerged as a research hotspot, unveiling complex bidirectional communication between bone and brain. Studies indicate that bone and brain can influence each other’s homeostasis via multiple pathways, yet there is a dearth of systematic reviews in this area. This review comprehensively examines interactions across three key areas: the influence of bone-derived factors on brain function, the effects of brain-related diseases or injuries (BRDI) on bone health, and the concept of skeletal interoception. Additionally, the review discusses innovative approaches in biomaterial design inspired by bone-brain interaction mechanisms, aiming to facilitate bone-brain interactions through materiobiological effects to aid in the treatment of neurodegenerative and bone-related diseases. Notably, the integration of artificial intelligence (AI) in biomaterial design is highlighted, showcasing AI’s role in expediting the formulation of effective and targeted treatment strategies. In conclusion, this review offers vital insights into the mechanisms of bone-brain interaction and suggests advanced approaches to harness these interactions in clinical practice. These insights offer promising avenues for preventing and treating complex diseases impacting the skeleton and brain, underscoring the potential of interdisciplinary approaches in enhancing human health.

## Introduction

In the complex physiological system of the human body, the skeleton, as the structural framework of the body, and the brain, as its cognitive center, are intricately linked to the maintenance of overall health.^1,2^ Recent studies have increasingly revealed that the skeleton and brain can interact through bone-derived factors (such as OCN,^3^ LCN 2,^4^ SOST^5^) or neuro-derived factors (such as leptin,^6^ NPY,^7^ 5-HT^8^) and extracellular vesicles (EVs).^9–11^ In clinical practice, many complex diseases and symptoms are not merely the result of isolated issues within the skeletal or brain but rather the outcome of interactions between the two.^12^ A singular focus on either bone theory or brain theory often fails to provide a comprehensive explanation or an effective treatment approach. For instance, patients with osteoporosis frequently experience cognitive decline, such as memory loss and difficulty concentrating.^13^ Focusing solely on bone metabolism fails to explain cognitive impairments, while examining the brain alone cannot account for decreased bone density. Biomaterials have been extensively applied in both the bone and brain fields independently.^12,14–18^ However, how to integrate these materials to simultaneously support both the skeleton and brain within the context of bone-brain interaction remains an underdeveloped area. Interestingly, Liu group discovered that biomaterials interact with the tissue microenvironment, performing multiple biological functions that significantly advance therapeutic outcomes.^19–22^ They defined this as “materiobiology”.^23^ Accordingly, this review summarizes the current research on the mechanisms of bone-brain interaction, aiming to integrate these insights with materiobiology to design biomaterials capable of cross-organ regulation between bone and brain, addressing complex clinical challenges in bone-brain disorders.

Following the recognition of bones as the largest endocrine organ in the human body, the interaction between the skeletal system and the brain has garnered significant research attention.^1^ Beyond the brain’s regulation of bone physiology, bone-derived factors such as fibroblast growth factor 23,^1^ bone morphogenetic proteins,^24^ osteopontin (OPN),^25^ and osteocalcin (OCN)^26^ have been shown to influence brain functions including development, learning, memory, and emotional regulation through pathways like blood circulation and neurotransmitter modulation.^27^ These factors also play pivotal roles in the pathogenesis of BRDI, offering new perspectives on their complexity. Conversely, BRDI such as Parkinson’s disease (PD), Alzheimer’s disease (AD), and depression has profound effects on bone health.^28,29^ Motor dysfunction, neurotransmitter imbalances,^30^ and chronic inflammation^29,31^ commonly observed in these conditions disrupt bone cell activity and metabolic homeostasis, increasing the risk of osteoporosis and fractures.^32^ Understanding the bidirectional mechanisms of bone-brain interactions is critical for developing therapeutic strategies that address both brain and skeletal disorders.

Skeletal interoception, the skeleton’s ability to sense and respond to internal and external stimuli, represents a fundamental aspect of bone-brain interactions.^33^ Acting as both a mechanosensitive and metabolic sensor, the skeletal system conveys critical feedback to the brain, influencing neural responses and systemic health.^34^ Recent research highlights the involvement of key molecules like prostaglandin E2 (PGE2) in mediating this process, linking mechanical loading, sensory signaling, and central regulation of bone homeostasis.^35^ This emerging field not only provides insights into the skeleton’s role in adapting to mechanical and metabolic demands but also reveals potential pathways for addressing conditions such as arthritis, spinal degeneration, and bone loss in microgravity.^36–38^ By exploring skeletal interoception’s mechanisms and therapeutic potential, we can unlock new strategies for treating neuro-skeletal disorders.

The challenge arises because the skeleton and brain differs significantly in function, with each having complex and interdependent biological mechanisms. The skeleton primarily provides structural support and mechanical functions, while the brain is responsible for information transmission and regulatory functions. Designing a biomaterial that is suitable for both systems requires the integration of multiple functional properties, such as osteoconductivity, osteoinductivity, neuroconductivity, and neuroprotection. This multifunctional integration presents significant design challenges, making it difficult to balance the different requirements, often leading to compromises in performance in one area or another. Furthermore, materials for bone and brain repair are typically developed within different engineering and biological domains. For example, bone repair materials often focus on mechanical strength and osseointegration, while brain repair materials emphasize softness, biocompatibility, and support for nerve regeneration.^39–42^ Combining the advantages of these two types of materials to design new composite materials requires crossing traditional disciplinary boundaries. This not only demands that researchers possess broad interdisciplinary knowledge but also necessitates the development of new approaches to material design and fabrication. The complexity of such cross-disciplinary design increases the challenges in materials research and development. Unlike conventional approaches that focus solely on materials design for either bone or brain, this paper highlights the interactive pathways between bone and brain, introducing potential design strategies for materials that could be applied in bone-brain interaction under the framework of materiobiology.^23^ Additionally, it proposes a concept for AI-assisted biomaterial design, specifically tailored for bone-brain interaction.

## How bone shapes the brain

Bone cells, such as osteocytes and osteoblasts can secrete various bone-derived factors to regulate bone metabolism.^43^ As research in osteology and neuroendocrinology progresses, the mechanisms by which the skeleton influences CNS health are gradually being uncovered.^44,45^ Furthermore, aging increases the permeability of the brain blood barrier (BBB), allowing more bone-derived factors to enter the brain from the periphery.^46^ These factors regulate neuronal survival and growth, synaptic plasticity, and neurotransmitter secretion within the brain, thereby influencing cognitive functions, memory, and learning abilities.^47^

### Sclerostin

**Fig. 1:**
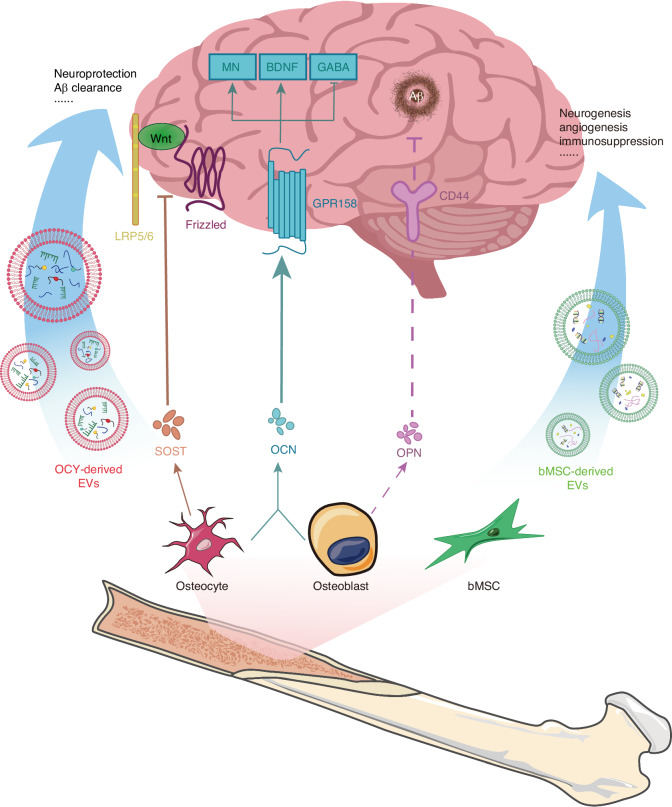
Regulation of bone-derived factors and EVs on brain health. LRP5/6: lipoprotein-receptor-related protein-5 or -6, OCY osteocyte, MN monoamine neurotransmitter, GABA gamma-aminobutyric acrid, GPR 158 G protein-coupled receptor 158

Sclerostin (SOST), primarily produced by osteocytes, is a secreted glycoprotein encoded by the *Sost* gene, with a molecular weight of approximately 22 kD. The canonical WNT/β-catenin pathway is a crucial regulatory pathway for bone formation. The WNT family of glycoproteins activates frizzled and LPR5/6 as co-receptors, stabilizing β-catenin, which in turn induces the transcription of genes involved in osteoblast differentiation and promotes bone formation. SOST acts as an antagonist of the WNT/β-catenin signaling pathway.^48^ SOST exerts its inhibitory effect by binding to LPR5/6, thereby preventing their interaction with the frizzled co-receptors^49^ (Fig. 1). SOST negatively regulates bone formation by antagonizing the WNT/β-catenin signaling pathway. The absence of SOST leads to sclerosteosis, characterized by high bone mass. Additionally, as a bone-derived factor, SOST also has regulatory effects on various other organs. Moreover, SOST also exerts regulatory effects on numerous other organs.^50^ For example, a lack of SOST affects the production of B lymphocytes and bone marrow cells, along with other changes within the bone marrow cavity that may impact hematopoiesis.^51,52^ Additionally, patients with chronic kidney disease exhibit elevated levels of SOST in their serum.^53^ In the brain, SOST advances the progression of AD through this pathway. Research indicates that the activation of the WNT/β-catenin signaling pathway has neuroprotective effects against amyloid-beta (Aβ) peptide toxicity, and it is also involved in Tau phosphorylation and the processes of learning and memory.^54^ The accumulation of Aβ plaques and the aggregation of Tau protein are considered two core characteristics of AD. In 2024, Jiang group discovered the molecular mechanism by which osteocyte-derived SOST regulates cognitive decline in AD through the WNT/β-catenin pathway.^55^ The study found that during aging and the progression of AD, osteocyte-derived SOST can cross the BBB in aged mice and bind to LPR6 receptors on neurons. This interaction leads to dysfunction of the WNT/β-catenin pathway and, through LPR6/β-catenin/β-secretase signaling, increases Aβ production, thereby exacerbating cognitive impairment associated with aging and AD progression. Interestingly, Dickkopf-related protein 1 (DKK1), which is also an antagonist of the WNT/β-catenin signaling pathway, has significant effects on both the brain and bones. DKK1 is considered a contributing factor to pathological bone necrosis, and thus, the use of DKK1 antibodies is seen as a potential treatment. Additionally, DKK1 causes synaptic degradation and neuronal apoptosis. Its high expression can even cross the BBB, which, in elderly individuals with a highly permeable BBB, undoubtedly exacerbates the progression of AD.^56^ Thus, it is evident that the WNT signaling pathway is a crucial link between the brain and bones. Researching the deep interactions between the brain and bones through the WNT signaling pathway holds great potential.

### Osteopontin

OPN is encoded by a single-copy gene and is expressed in cells of various tissues, including bone, dentin, cementum, hypertrophic cartilage, kidney, brain, endometrial glandular cells derived from bone marrow, uterine and decidual vascular tissues, trophoblasts, inner ear ganglia, mammary gland, salivary and sweat glands, bile and pancreatic ducts, distal renal tubules, and specialized epithelial cells in the intestine and brain.^57^ It is also expressed in activated macrophages and lymphocytes, but it is primarily produced by osteoblasts and osteocytes in the bone.^58^ Although the OPN protein in mammals and birds contains a similar number of amino acids, the modifications of OPN differ among species, leading to unusual behavior in SDS-PAGE analysis. As a result, the molecular weight of OPN varies between species, ranging approximately from 44 kD to 75 kD.^57^ The highly phosphorylated nature of OPN endows it with strong mineral-binding properties. William N. Addison discovered in MCETE-E1 cell cultures that the phosphorylation of OPN facilitates its interaction with calcium in hydroxyapatite, thereby stabilizing the mineral precursor and reducing mineral formation.^58^ Tissue-nonspecific alkaline phosphatase can dephosphorylate OPN, reversing this effect. The impact of OPN on the CNS is even more complex. It is present in nearly all neurons, glial cells, and immune cells.^59^ Numerous studies have found that OPN has neuroprotective effects and can promote the repair of conditions such as stroke, cerebral ischemia, and Traumatic Brain Injury (TBI).^60^ Robert Meller et al. discovered in a stroke model that OPN promotes the phosphorylation of Akt, achieving neuroprotection through the PI3K/Akt pathway.^25^ The neuroprotective effect induced by OPN was blocked by the PI3K inhibitor LY294002. Additionally, OPN binds to integrins and CD44 (Fig. 1), facilitating neural signal transmission, supporting the health and integrity of the basement membrane, and promoting the migration, proliferation, and survival of primary astrocytes under stress conditions.^61,62^ However, OPN does not seem to have solely positive effects in the brain. Research has found that OPN is highly transcribed in the lesions and spinal cord of rat models of multiple sclerosis with experimental autoimmune encephalomyelitis (EAE).^63^ OPN knockout mice exhibited milder EAE symptoms. Subsequent research indicated that this effect is due to OPN-C, which is generated by thrombin cleavage.^64^ OPN also mediates the formation of corpora amylacea in the hippocampus. Tae-Ryong Riew et al. discovered that in the late stages of cerebral ischemia, astrocytes synthesize and secrete OPN, which then precipitates as a key component of corpora amylacea.^65^ This finding is in stark contrast to the numerous studies that have demonstrated the neuroprotective role of OPN in AD. These opposing effects are believed to be caused by several factors: (1) the wide variety of cell types that express OPN; (2) the enzymatic cleavage of OPN into different active fragments, each with distinct functions; (3) the presence of OPN in various locations, where it exerts its effects through multiple signaling pathways.^66^

### Osteocalcin

OCN, also known as bone γ-carboxyglutamic acid protein or BGP, is encoded by the Bglap1 and Bglap2 genes. It is exclusively expressed by osteoblasts and contains 46-50 amino acid residues (Mr = 5210-5889), depending on the species. OCN is a Ca^2+^ binding protein abundantly present in the organic matrix of bone tissue, dentin, and other mineralized tissues.^67^ In the human body, OCN exists in two different forms, differentiated by their degree of carboxylation.^68^ One form is fully carboxylated osteocalcin (tOCN), which is the most abundant non-collagenous protein in the bone extracellular matrix (BECM). The other form is undercarboxylated osteocalcin (uOCN), which is present in systemic circulation at concentrations of nanograms per milliliter, similar to the levels of most hormones. Initial studies focused on tOCN’s effects on bone metabolism within the bone matrix. However, they did not find any regulatory role for OCN in bone mineralization. However, when researchers speculated about OCN’s potential effects outside the skeleton, they discovered that it could regulate various aspects such as energy metabolism, reproduction, and neural development through endocrine pathways. Studies on OCN-deficient mice revealed that peripheral OCN can cross the BBB and bind to multiple brain regions, including the hippocampal CA3, the ventral tegmental area, and the dorsal raphe nucleus.^68^ Khrimian confirmed through genetic, electrophysiological, molecular, and behavioral analyses that GPR158 is the receptor for OCN in the hippocampal CA3 region.^69^ They found that exogenous OCN can promote the synthesis of monoamine neurotransmitters (including serotonin, dopamine, and norepinephrine) and brain-derived neurotrophic factor (BDNF) through this pathway, while inhibiting the synthesis of the neurotransmitter GABA, thereby improving cognitive function.^60,68,70^ Interestingly, OCN can also influence the development and function of the embryo’s brain through maternal effects. Oury discovered that during pregnancy, maternal OCN can cross the placenta and prevent neuronal apoptosis in the embryo, which is not yet capable of synthesizing osteocalcin.^71^ Additionally, acute high-intensity interval exercise (HIIE) interventions have been shown to increase the levels of both tOCN and uOCN in the body. uOCN can cross the BBB and further enhance the expression of BDNF mRNA and protein in the brain, thereby improving cognitive abilities and delaying CNS aging (Fig. 1). Unfortunately, the effects of HIIE intervention are not permanent, as the levels of serum OCN and uOCN do not show significant changes after four weeks. Therefore, long-term exercise can regulate and prevent age-related cognitive decline by maintaining elevated uOCN levels.^72,73^

### Exosomes

Exosomes are a type of extracellular vesicle that contain a variety of proteins, lipids, and nucleic acids.^74^ Exosomes have been found to be secreted by various cell types, including lymphocytes, dendritic cells, platelets, mast cells, neurons, macrophages, mesenchymal stem cells (MSCs), and intestinal epithelial cells, playing a crucial role in intercellular communication.^75^ Research has shown that in bone tissue, bone marrow stromal cells (bMSC), osteoclasts, osteoblasts, and osteocytes can all release exosomes, which regulate bone remodeling and skeletal diseases. A typical example is osteoclasts secreting exosomes containing RANK, which can bind to RANKL on osteoblasts, thereby influencing the process of bone remodeling and repair.^76^ Recent studies have increasingly supported the hypothesis that bone-derived exosomes can affect the brain. The exosomal membrane allows them to cross the BBB, providing a structural basis for using exosomes to treat brain diseases.^77^ Additionally, stem cell-derived exosomes play a crucial role in affecting BRDI. MSCs, which are an important type of stem cell capable of secreting exosomes, are abundantly present in the bone marrow. This provides a tissue basis for the influence of bone-derived exosomes on the brain.

Stroke, as a highly disabling acute disease, causes severe neurological damage and presents significant challenges in later recovery. In stem cell therapies used for stroke treatment, MSC-derived exosomes have been increasingly recognized as a key factor in restoring neural activity^78^ (Table 1). Therefore, stroke is a significant target for exosome therapy. Research indicates that exosomes contribute to stroke recovery in multiple ways. In a rat model of cerebral hemorrhage, MSC-derived exosomes have been found to promote neurogenesis, vascular remodeling, and myelin regeneration, significantly improving the rats’ behavioral and cognitive abilities.^79^ Li provided an explanation for the angiogenic capacity of bMSC-derived exosomes from the perspective of the chemokine CXCR4.^80^ Exosomes from bMSCs with high expression of the chemokine receptor CXCR4 promote the proliferation, angiogenesis, and anti-apoptotic effects of microvascular endothelial cells in stroke rats through the Wnt-3a/β-catenin pathway. These exosomes contain miRNAs that play significant roles in cell function, disease, and immune regulation.^81^ Studies have shown that miRNAs can mediate the therapeutic effects of MSC-derived exosomes.^82^ For instance, in the hippocampal tissue of depressed rats, miRNA-26a is upregulated by bMSC-derived exosomes, increasing SOD levels in the hippocampus of depressed rats, and reducing the levels of MDA, LDH, TNF-α, and IL-1β. This promotes hippocampal neuron proliferation and inhibits apoptosis.^83^ Exosome therapy alone is also an effective approach for treating neural injuries.^84^ MiRNAs within exosomes are believed to improve hypoxia in neural cells and promote vascular regeneration, making them a promising treatment for ischemic stroke, the most common type of stroke. Hu^85^ found that bMSC-derived exosomes can upregulate the expression of pro-angiogenic factors such as VEGF, VEGFR2, Ang-1, and Tie-2 through miR-21-5p, thereby improving angiogenesis in mice with ischemic stroke. VEGF and VEGFR2 promote endothelial cell migration and the formation of blood vessels, while Ang-1 and Tie-2 maintain the stability of the vascular network.^86,87^ An exciting finding in this study was that rat bMSC-derived exosomes can promote the proliferation of human umbilical vein endothelial cells in vitro.^85^ This implies the superior immunocompatibility of cross-species bMSC-derived exosomes, indicating that cross-species bMSC-derived exosome therapy is a promising strategy for treating stroke.

**Table 1:** Bone derived exosomes treat BRDI

Origin	Animal model	Brain related diseases or injuries	Cargo	Function	Ref.
bMSC	Rat	intracerebral hemorrhage	–	Promote neurovascular and white matter remodeling	^79^
bMSC	Rat Mice	Stroke	CXCR4 miR-21-5p	Promote microvascular endothelial cell proliferation, angiogenesis, and anti-apoptosis	^80,85^
bMSC	Rat	SAH	miR-129-5p	Anti-inflammatory and anti-apoptotic	^89^
bMSC	Rat	Depression	miR-26a	Inhibit apoptosis and promote neuronal regeneration	^83^
bMSC	Rat Mice	TBI	- miR-181b	Promote neurovascular, Anti-inflammatory	^90,266^
Young osteocyte	Mice	AD	–	Neuroprotection, Aβ clearance, and enhanced cognitive function	^202^

In another type of hemorrhagic stroke, subarachnoid hemorrhage (SAH), bMSC-derived exosomes carrying miRNAs have also been proven to have positive effects. SAH leads to increased intracranial pressure and reduced cerebral blood flow, resulting in global cerebral ischemia. This condition causes BBB disruption, cell apoptosis, and severe brain inflammation.^88^ Therefore, Xiong investigated the effects of exosomes on early brain injury following SAH from the perspectives of apoptosis and inflammation.^89^ They found that local injection of bMSC-derived exosomes into SAH rats led to increased expression of miR-129-5p in the hippocampus of the SAH rats. With the increased expression of miR-129-5p, the levels of the inflammatory mediator high-mobility group box 1 were reduced at both the RNA and protein levels.^89^ Additionally, the expression of TNF-α and p53 also decreased, resulting in the suppression of inflammation and apoptotic cascades in the brain. They also speculated on the source of the increased miR-129-5p expression in the hippocampus of SAH rats following local injection of bMSC-derived exosomes. Firstly, they considered that the injected exosomes themselves could be a significant source of miRNA. Secondly, they hypothesized that bMSC-derived exosomes might carry certain substances that stimulate the expression of miRNAs in neighboring tissues. Whether bMSC-derived exosomes have a direct impact on apoptotic cells or can rescue them from apoptosis is also a question worth exploring.

**Table 2:** Material design strategies for bone-brain interactions

Materials	Functional structures	Characteristics	Ref.
RAPA@tRPCs	Stroke-homing peptide	Stroke targeting	^216^
9Phe@T7-LNPs	T7 stroke targeting peptide, liposome	Stroke targeting, BBB penetration	^267^
FTY@Man NPs	PLGA-PEG skeleton	BBB penetration	^268^
Glucose-coated Gold NPs	Glucose coating	BBB penetration	^269^
PEGylated LNs	PEGylation	Opsonization and phagocytosis reduction, brain targeting, neurotoxicity reduction	^270^
FiGNPs	Fucoidan	BBB endothelial P-selectin targeting	^271^
MM@MnO2-Au-mSiO2@Cur NPs	Macrophage membrane	Inflammatory targeting	^272^
RBC-coated MSNs	RBC membrane coating	Prolong the presence of the drug in the bloodstream, modifiability	^232^
RBCM-NPs	RBC membrane coating, Anti-EGFR-iRGD	Modifiability, tumor targeting	^228^
NPs@NEs, SPC_Fe_/siP	Neutrophil delivery	Bone marrow targeting	^240^
		Inflammation targeting	^273^
490BP-C14 LNP	Bisphosphonate		
BTNPs	Alendronate	Bone targeting	^274–276^
ZA-m7G Runx2 mRNA-LNPs	Zoledronic acid-DSPC		

Additionally, exosomes derived from bone marrow mesenchymal stem cells can inhibit neuroinflammation by modulating glial cell phenotypes and promoting neural injury repair (Fig. 1). This has shown positive effects in a range of BRDI, including stroke. Based on microRNA sequencing, Wen further investigated miRNAs closely associated with neuroinflammation.^90^ They found that miR-181b may regulate the phenotypic changes of microglia through the IL-10/STAT3 pathway, thereby inhibiting neuroinflammation and cell apoptosis. Neuroinflammation is also a significant factor threatening the neural health of AD patients. As Aβ accumulates, microglia and astrocytes become activated and release inflammatory cytokines, exacerbating neuroinflammation.^91,92^ Studies have found that intraventricular injection of bMSC-derived exosomes can reduce the expression of BACE, Aβ_1-42_, and p-tau in the hippocampus of AD mice, while upregulating the expression of BDNF.^92^ This treatment alleviates neuroinflammation, mitigates neuropathological symptoms of AD, and promotes neuronal regeneration. Additionally, the impact of exercise on the brain has also garnered significant attention. Jiang^93^ studied the potential impact of exercise on brain injury from the perspective of the synergistic effects of bMSC-derived exosomes and exercise in a rat model of stroke. Their research found that both bMSC-derived exosomes and treadmill exercise can mediate neural remodeling after ischemic stroke through the JNK1/c-Jun pathway. In the combined exercise and bMSC-exos group, ischemic stroke mice exhibited higher levels of JNK and c-Jun phosphorylation. In the combined exercise and bMSC-exos group of ischemic stroke mice, the expression levels of synaptophysin, PSD-95, MAP-2, GAP-43, and NF-200 were elevated, promoting synapse formation and axon regeneration. Additionally, this combination inhibited neuronal apoptosis and reduced infarct volume, thereby enhancing cognitive recovery in ischemic stroke mice. This study provides new insights into the synergistic effects of treadmill exercise and bMSC-derived exosomes.

## How the brain influences bone

The brain, as one of the most complex organs in the human body, plays a crucial role in regulating bone health.^94^ Recent studies have shown a close connection and mutual influence between the CNS and the skeleton. This emerging field is referred to as the brain-bone axis.^95^ Through regulation by the nervous system, the brain can influence bone metabolism and regeneration, thereby exerting either positive or negative effects on bone health.^96^ Research indicates that certain BRDI can significantly impact bone health, including but not limited to osteoporosis, fracture healing, and musculoskeletal pain syndromes.^97^ These diseases may directly or indirectly alter bone metabolism and structure through their effects on the nervous system, thereby affecting an individual’s overall health. This article reviews the associations between various BRDI and bone health. Unfortunately, the underlying mechanisms of brain regulation on bones remain largely unexplored. Further research is needed to understand how the brain regulates bone tissue through neural connections and how bones, in turn, influence the brain via hormones and other pathways.^98^ Future research should focus on exploring the molecular, genetic, and physiological foundations of the brain-bone axis, as well as investigating the future development directions of this emerging field. Through deeper exploration, we aim to uncover the regulatory mechanisms of the brain on bones, providing a more comprehensive and profound scientific basis for the prevention and treatment of related diseases.

### Traumatic brain injury

**Fig. 2:**
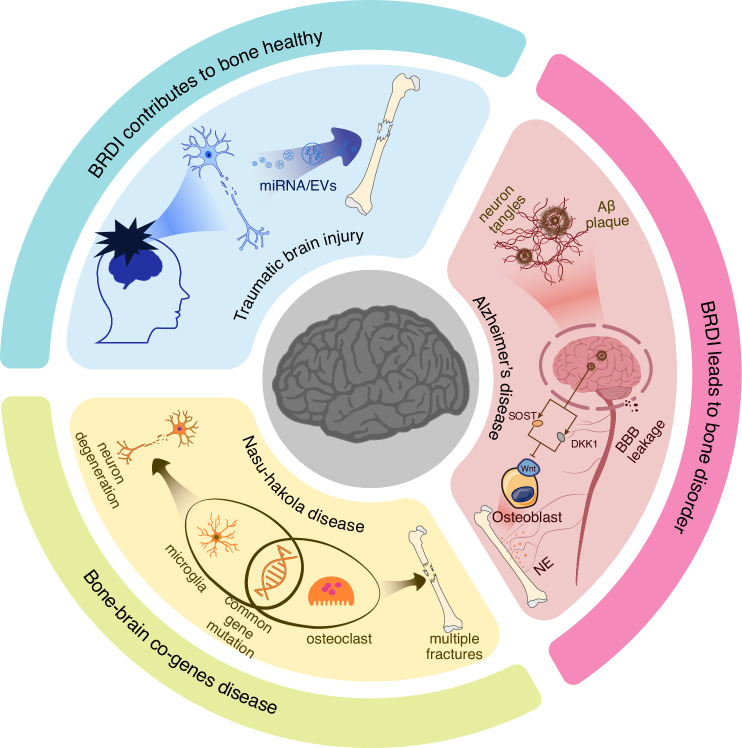
The impact of BRDI on bone metabolism. BRDI such as TBI, have a positive impact on bone homeostasis. Damaged nerve cells can release EVs carrying miRNAs that promote fracture healing. In contrast, BRDI like AD negatively affects bone metabolism. The accumulation of Aβ plaques in the brains of AD patients leads to neuron tangles and disrupts the BBB, triggering brain inflammation. At the same time, it increases the expression of SOST and DKK1, both of which inhibit osteogenesis by suppressing the Wnt signaling pathway. This, in turn, activates the SNS, leading to increased secretion of NE and subsequent bone loss. Additionally, bone-brain co-genes diseases, exemplified by Nasu-Hakola disease, provide a new perspective on the genetic underpinnings of bone and brain disorders

TBI has become a significant global health and socioeconomic issue.^99^ The widespread trends of aging populations and the increasing prevalence of motor vehicles have led to a rising incidence of TBI. TBI is characterized by vascular dysfunction and hypoxia, followed by subsequent ischemia.^100^ Therefore, in addition to the direct trauma to the brain caused by TBI, the resulting neural damage and intracerebral inflammation may contribute to the development of neurological diseases such as AD, PD, and depression. Interestingly, patients with TBI combined with long bone fractures often experience faster fracture healing compared to those with isolated fractures, with increased skeletal formation at the fracture site and even instances of heterotopic ossification. A substantial body of preclinical research has reached a consensus: TBI accelerates fracture healing.^101^ Extensive research into the mechanisms has been conducted, with preliminary hypotheses suggesting that time-dependent changes in the microenvironment following brain injury, along with the disruption of the BBB, increase the exchange of substances between the brain and peripheral tissues. In recent years, exosomes have emerged as a potential answer to the question of how TBI accelerates fracture healing (Fig. 2). Yang found that exosomes extracted from the plasma of a mouse model with TBI and tibial fractures can promote the proliferation of MC3T3-E1 cells.^102^ The study also revealed the significant role of miRNAs carried by neurogenic exosomes in bone remodeling. Building on previous research, they confirmed the ability of miR-22-3p, miR-34a-5p, and miR-378a-3p to promote osteoblast differentiation, even though they were unable to determine the exact source of the exosomes or the mechanisms by which these miRNAs enhance osteogenesis.^102^ Similarly, Guo identified four long non-coding RNAs (lncRNAs)—ENSG00000278905 (AC106818.1), ENSG00000240980 (AC093904.1), ENSG00000255670 (AC007619.1), and ENSG00000196634 (LUADT1)—which also play a role in fracture healing under TBI conditions through their transport via EVs.^103^ Xia addressed these questions in their research by analyzing clinical samples and a rat model of TBI combined with fractures.^104^ Proteomic analysis revealed that the A2M protein, a marker of neuronal injury, was elevated in EVs from the plasma following TBI. This finding confirmed that the exosomes promoting fracture healing originate from damaged neurons. Additionally, they provided an explanation for how EVs target the fracture site. The study found that the membrane surface protein FN1 was elevated in the plasma EVs of TBI rats, guiding the EVs to target osteoprogenitor cells and bone. Furthermore, the study identified that miR-328a-3p and miR-150-5p within these EVs promote bone formation by downregulating FOXO4 and CBL. Although there is still a lack of molecular-level understanding, particularly concerning cytokines, of how TBI promotes fracture healing, the aforementioned studies provide valuable insights for the development of biomaterials. Mimicking EVs with liposomes or bioaffinity microsphere materials can be modified with specific membrane proteins to achieve precise targeting. Additionally, since many factors, such as those in the interleukin family, have time-dependent effects, designing appropriate controlled-release mechanisms to regulate the release of internal factors or drugs within the materials is also crucial.

### Alzheimer’s disease

AD is one of the most common neurodegenerative diseases. There is no definitive understanding of the mechanisms underlying its onset. Currently, AD is characterized by the presence of Aβplaques and neurofibrillary tangles containing tau protein.^105^ The former is believed to induce the pathological spread of tau protein through an unknown mechanism.^106^ PD is the second most common neurodegenerative disease after AD. Autopsy reports show that similarly, 60%–80% of PD patients also exhibit pathological phenomena such as Aβ accumulation, tau protein hyperphosphorylation, and neurofibrillary tangles.^107,108^ Recent studies have found that elderly individuals with AD have lower bone density compared to healthy elderly individuals. This undoubtedly increases the risk of fractures in AD patients who already suffer from cognitive, visuospatial, and executive impairments, further limiting their mobility.^105,109^ The impact of AD on the increased fracture risk in patients is multifaceted and complex. Starting with the characteristics of AD, the presence of Aβ alone has multiple effects on bone metabolism. Aβ itself is a protein that regulates synaptic activity and neuron survival. It is produced by the sequential cleavage of amyloid precursor protein (APP) by β- or γ- secretase. However, the deposition of Aβ in the brain leads to neurotoxicity, particularly in the context of aging.^110^ McLeod discovered Aβ deposition on the endosteal and periosteal surfaces of the ulna in adult rats.^111^ Subsequently, Li and colleagues found that Aβ42 and APP are prominent features in osteoporotic sites in both humans and rats.^112^ Furthermore, the study indicated that human Aβ42 can exacerbate osteoporosis by promoting osteoclast fusion, even though Aβ42 does not affect the viability and number of osteoclast cells.^112^ Shortly after that, they provided further explanation for the promoting effects of Aβ on osteoclasts. Their research demonstrated that Aβ enhances RANKL-induced osteoclasts activation through the degradation of IκB-α, ERK phosphorylation, and calcium oscillation signaling pathways.^113^ Additionally, Xia and colleagues demonstrated that APP can inhibit the differentiation of osteogenic lineage cells, which could be a significant reason for bone loss in AD patients.^114^ In addition to its direct effects on bone resorption and formation, Aβ deposition also impacts the Wnt/β-catenin signaling pathway in AD patients. As previously mentioned, the Wnt/β-catenin pathway has neuroprotective effects, and its impairment can accelerate the progression of AD. SOST and DKK1, as inhibitors of this pathway, pose a serious threat to neuronal health when expressed in the brain. Aβ can excessively activate DKK1 expression, thereby inhibiting the Wnt/β-catenin signaling pathway.^115^ This results in hindering the growth and differentiation of neural cells and disrupting neuronal plasticity in the brain. For bones, this leads to increased bone resorption and exacerbates bone loss (Fig. 2). Aβ‘s excessive activation of DKK1 expression also leads to the hyperphosphorylation of tau protein, promoting tau protein aggregation, which is the second hallmark of AD.^116^ In addition to the aforementioned effects, AD patients typically experience chronic inflammation, and the inflammatory environment in their bodies may accelerate systemic bone loss. Moreover, the brain regulates bone metabolism through sensory-sympathetic/parasympathetic neural circuits. Increased sympathetic nervous tension enhances osteoclast activity, promoting bone resorption.

In summary, both AD and osteoporosis are systemic degenerative diseases with widespread pathogenic factors and complex mechanisms of influence. Although the exact pathogenesis of AD has not been fully elucidated, current understanding suggests that biomaterial-based interventions might be inspired by strategies to clear Aβ from the brain or to repair the BBB to prevent excessive peripheral Aβ from entering the CNS, or at least to neutralize Aβ.

### Depression

With the development of the economic and social landscape, there has been a growing focus on mental health. Depression is a common mental disorder, with approximately 280 million people affected worldwide, according to WHO statistics. The link between depression and osteoporosis, both of which have high prevalence rates, has been a topic of significant interest. However, this relationship remains controversial. Most official publications, such as those from the National Institutes of Health, the National Osteoporosis Foundation, the National Osteoporosis Society (UK), and Osteoporosis Canada, do not fully recognize depression as a risk factor for low bone mass and osteoporosis due to a lack of sufficiently convincing research evidence.^117^ In 2001, Giovanni and colleagues published the first review on the relationship between depression and osteoporosis, indicating a strong connection between the two conditions.^118^ Subsequent research found that patients with major depressive disorder exhibit significantly elevated levels of bone resorption markers.^119^ Bab and colleagues demonstrated that the loss of bone mass and structure in chronic mild stress mice, an established rodent model of depression, is primarily due to decreased bone formation.^117^ Depression continuously activates the hypothalamic-pituitary-adrenal axis by stimulating the release of corticotropin-releasing hormone from the hypothalamus through neural connections in the prefrontal cortex, hippocampus, amygdala, and hypothalamus, leading to elevated cortisol levels.^120^ Interestingly, Cushing’s syndrome is a typical disease characterized by osteoporosis symptoms resulting from elevated cortisol levels. Unlike depression, patients with Cushing’s syndrome exhibit significantly higher cortisol levels, including elevated plasma and urinary cortisol.^121^ A study has indicated that patients with Cushing’s syndrome caused by adrenal adenomas exhibit abnormally elevated cortisol levels, along with bone loss and osteoporosis. The study also identified a condition known as mild autonomous cortisol secretion, which features cortisol elevations more closely resembling those in depression. While patients with this condition experience only slight cortisol excess and do not display overt Cushing’s symptoms, it nonetheless significantly impacts bone health.^122^ Relevant studies exploring the mechanisms by which cortisol induces osteoporosis have found that cortisol can inhibit bone remodeling by suppressing both bone formation and bone resorption. In rats, cortisol has been found to suppress osteoblast expression of IGF I at the transcriptional level through exon 1 of the IGF I gene.^123^ However, high concentrations of cortisol can directly impact the RANK/RANKL/OPG system by increasing RANKL expression, thereby promoting osteoclast differentiation and activity.^124^ Additionally, a clinical study on competitive cyclists suggests that elevated cortisol levels may affect bone mineral absorption.^125^ Although this study does not conclude that the rise in cortisol before and after cycling competitions reduces bone density, it suggests that long-term cycling may negatively impact bone density. The study further recommends that cyclists increase calcium intake in their daily diet. This effect may relate to the duration of elevated cortisol levels; conditions such as depression and mild autonomous cortisol secretion, where cortisol remains chronically elevated above physiological levels, are more likely to lead to bone loss.

Depression, especially major depressive disorder, is often accompanied by neuronal dysfunction, such as neurotransmitter imbalances and changes in neural plasticity. This may provide a crucial breakthrough in understanding the link between depression and osteoporosis. Recent studies on the brain-bone axis have shown that neurotransmitters can influence bone density. In addition to cortisol, levels of norepinephrine, a postganglionic sympathetic neurotransmitter, are also elevated in patients with depression.^117,126^ This elevation is generally considered a result of increased sympathetic nervous tension in depression. The ability of propranolol, an adrenergic receptor antagonist, to block bone loss induced by chronic mild stress, but not the depressive state itself,^117^ further supports the role of the sympathetic nervous system as a key efferent pathway in regulating bone metabolism activities. A recent study elucidated the cellular and molecular mechanisms by which propranolol enhances the anabolic effects of parathyroid hormone (PTH) on systemic bone loss following a fracture.^127^ Fracture triggers increased sympathetic nervous tension, and norepinephrine (NE) released by the sympathetic nerves mediates osteoclast differentiation by promoting the secretion of RANKL from osteoblastic cells.^128^ Additionally, NE inhibits PTH-induced osteoblast differentiation by suppressing the expression of BMal1 and Runx2. Moreover, the binding of NE to β-adrenergic receptors (βAR) on osteocytes stimulates the release of neuropeptide Y, which locally inhibits the proliferation of osteoblasts. Propranolol enhances the osteogenic effects of PTH by inhibiting NE-activated βAR signaling. Similarly, from the perspective of neurotransmitters, Erdem and colleagues studied the effects of the selective serotonin reuptake inhibitor fluoxetine and the serotonin-norepinephrine reuptake inhibitor venlafaxine on bone defect healing.^129^ However, neither drug showed significant healing effects in a rat bone defect model. The impact of fluoxetine and venlafaxine on bone metabolism is a topic of debate. Given the complex etiology of depression and the unclear relationship between depression and bone loss, the effects of these antidepressants on bone defect healing can vary depending on factors such as timing and dosage, and may even be contradictory across different studies.

In summary, although significant progress has been made in the pathophysiological study of mental illnesses like depression, describing the mechanisms of depression remains a challenging task. This difficulty in mechanistic understanding also leads to challenges in accurately identifying and diagnosing depression, particularly mild depression.^130^ This poses significant challenges to studying the link between depression and bone loss, as it is difficult to distinguish mild depression, and for patients with severe depression, bone loss may seem insignificant. Research on the connection between depression and bone loss could become a classic topic within the brain-bone axis. This issue encompasses several aspects:

1. The impact of abnormal inflammation on bone metabolism in a depressive state.
2. Pathological changes in neurons lead to neurotransmitter abnormalities that impact bone metabolism.
3. Abnormalities in the neuroendocrine axis, such as the hypothalamic-pituitary-adrenal axis.

Additionally, as previously mentioned, the sympathetic nervous system as an efferent pathway can cause bone loss under depressive conditions. An interesting question arises: whether the afferent sensory nerves, which may experience altered self-perception in a depressive state, can affect central processing and subsequently influence the downstream efferent nervous system and bone metabolism balance. This could be a fascinating area of research within the brain-bone axis.

### Nasu-Hakola disease

Nasu-Hakola disease, also known as polycystic lipomembranous osteodysplasia with sclerosing leukoencephalopathy, is a rare autosomal recessive disorder characterized by osteolytic bone lesions and early-onset frontotemporal dementia. It is marked by progressive presenile dementia and bone cysts.^131,132^ The disease typically manifests in the third to fourth decade of life, leading to death within a few years.^133^ Clinically, Nasu-Hakola disease is typically divided into four stages^132,134,135^:1. Latent Phase: Early developmental period. 2. Osteolytic Phase: Usually begins in the thirties, characterized by multiple bone cysts and pathological fractures due to trabecular bone loss. 3. Neurological Phase: Typically starts in the forties, marked by frontal lobe syndrome and progressive dementia. 4. Dementia Phase: Features global aphasia, loss of motor abilities, and increased recurrent intake.

Therefore, Nasu-Hakola disease is a typical example of a disorder involving the bone-brain connection. Multiple case studies have shown that patients with Nasu-Hakola disease often develop multiple bone cysts or fractures around the age of thirty. Within a few years following the onset of fractures, these patients begin to exhibit cognitive decline and other neurodegenerative changes.^131,132,135^ Research indicates that mutations in the TREM2 gene are a significant cause of Nasu-Hakola disease.^136^ The TREM2-DAP12 signaling pathway in microglial cells is involved in cell survival, phagocytosis, and the actin reorganization required for cell activation and phagocytic activity. This pathway also plays a crucial role in osteoclast formation and the dynamic balance of bone remodeling. Loss of function in DAP12 or TREM2 in human peripheral blood mononuclear cells leads to inefficient and delayed RANKL-induced osteoclast differentiation.^137^ This explains why patients with Nasu-Hakola disease exhibit both skeletal disorders and neurodegeneration in the brain.

Although Nasu-Hakola disease is a rare disorder, studying its pathogenesis is of great significance for understanding the connection between bone and brain. The brain is a complex organ composed of various cell types, including neurons, microglia, and astrocytes. Beyond the commonly understood interactions between nerves and bones, the interactions involving other brain cells such as microglia, the brain microenvironment, and the broader context of brain-bone interactions should all be considered part of the bone-brain axis. In Nasu-Hakola disease, TREM2 gene mutations lead to dysfunction in both microglia and osteoclasts, which in turn damage neuronal health and result in cognitive decline (Fig. 2). Utilizing a microglia-free niche created by depleting microglia and subsequently performing microglia replacement therapy has emerged as a potential treatment strategy for diseases caused by microglial gene mutations.^138^ Yoo and colleagues transplanted TREM2+/+ CDMCs into TREM2−/− 5xFAD mice, an Alzheimer’s disease model.^139^ This microglia replacement therapy significantly improved the containment and phagocytosis of Aβ plaques by brain myeloid cells, alleviating AD symptoms. Moreover, the study demonstrated that the transplanted CDMCs in the microglia replacement therapy showed high levels of long-term cellular chimerism in both the peripheral system and the brain.^139^ Therefore, using microglia replacement therapy to transplant myeloid cells with healthy genes may be beneficial for treating Nasu-Hakola disease. Extending this approach, research and treatment of bone-brain related diseases could start effectively from the genetic intersections of these two organs. Certainly, numerous related studies have already been conducted. For instance, Jiang and colleagues explored how SOST influences AD progression through the Wnt/β-catenin pathway.^55^ Zeynep Okur and collaborators found that the BMP2-SMED1 signaling pathway controls the excitatory-inhibitory balance in neurons.^140^ Zhang’s team discovered that Dmp1+ astrocytes, a bone cell marker, can transfer mitochondria to brain endothelial cells to maintain BBB integrity.^141^ Similarly, bone cells can transfer mitochondria to transcortical vessels to promote angiogenesis.^142^

## Skeleton interoception

Bone-brain interactions rely not only on biochemical signaling and disease-induced changes but also on the skeleton’s capacity to sense and respond to internal and external stimuli—an ability termed skeletal interoception.^33^ Acting as a mechanosensitive and metabolic sensor, the skeletal system provides feedback to the brain, influencing neural responses and systemic health. Understanding skeletal interoception’s role in mediating energy metabolism and mechanical load adaptation sheds light on its integral function within the bone-brain axis and its potential therapeutic implications.

In the interoceptive system, sensory nerves-brain-sympathetic/parasympathetic nervous systems form a non-conscious neural signaling pathway.^33^ Under a brain-centered framework, sensory nerves transmit stimuli from peripheral organs to the brain for signal processing, a process termed the ascending interoceptive pathway.^143–146^ Conversely, the sympathetic/parasympathetic nerves deliver brain-processed instructions back to peripheral organs.^147^ Pain sensation represents a critical signal conveyed by sensory nerves, and as a result, current research on skeletal interoception predominantly focuses on bone diseases with pronounced pain symptoms, such as intervertebral disc degeneration and arthritis.^148^

PGE2 is a pivotal lipid molecule with diverse physiological functions. Its neuroactivating properties and bone-related roles make it a potential mediator of bone-nerve interactions.^149^ Cao and colleagues substantiates this hypothesis, demonstrating that PGE2 binds to EP4 receptors on sensory neurons, activating the cAMP signaling pathway.^150^ The cAMP produced subsequently activates protein kinase A (PKA), which induces the phosphorylation of cAMP response element-binding (CREB) protein in the ventromedial hypothalamic (VMH) nucleus. Phosphorylated CREB translocates into the neuronal nuclei within the VMH to regulate the expression of genes associated with sympathetic tone, resulting in the downregulation of sympathetic activity and promoting bone anabolism. This role of PGE2 as a signal in skeletal interoception also provides a mechanistic explanation for the paradoxical observation that higher PGE2 levels, despite its pro-osteogenic properties, are inversely correlated with bone mass.^151^

**Fig. 3:**
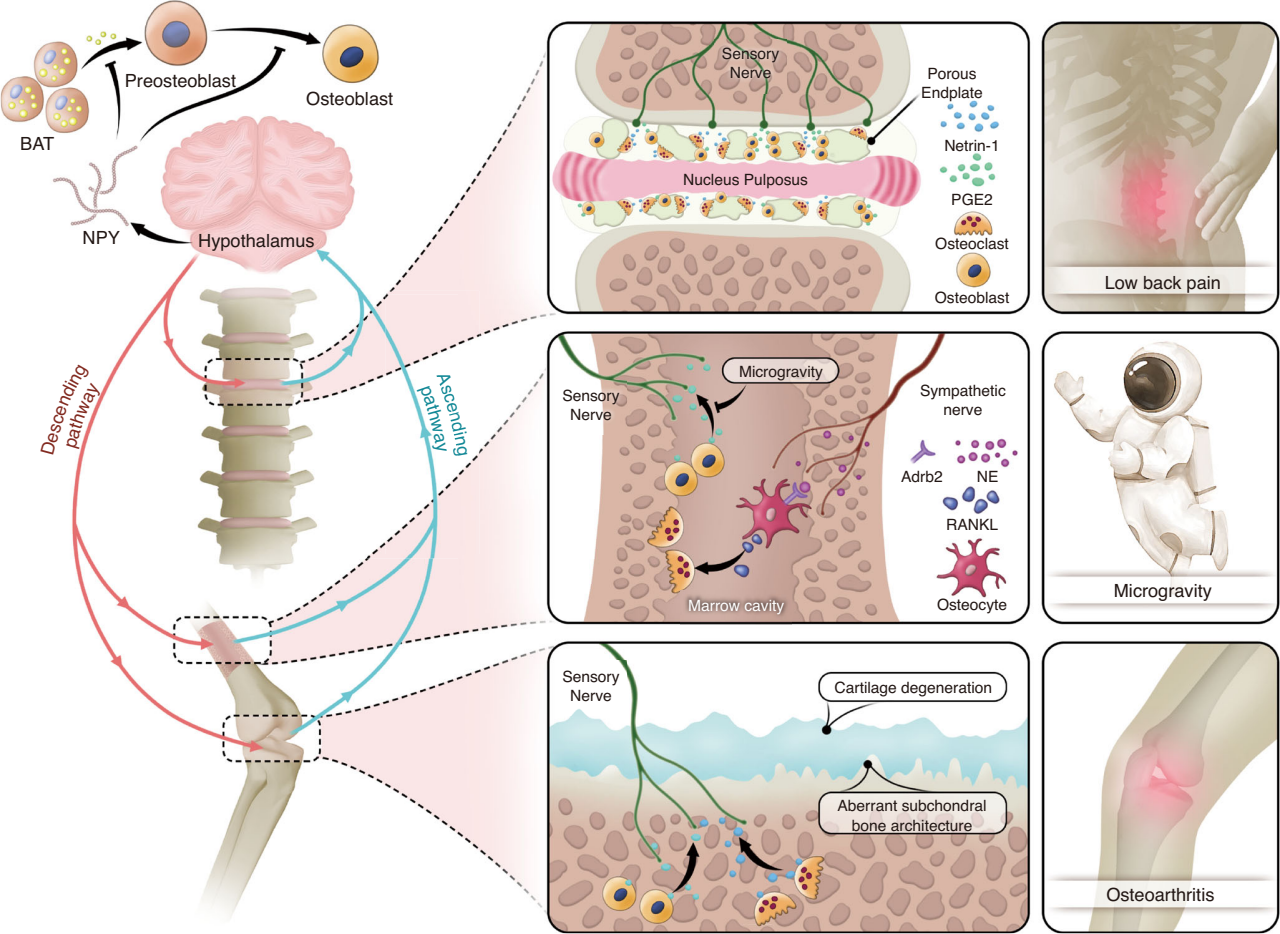
Mechanical influence and energy regulation mediated by skeletal interoception. Abnormal mechanical loading induces calcification of the intervertebral disc endplates and articular cartilage, as well as increased osteoclast activity. Osteoclasts in bone resorption lacunae secrete Netrin-1, which induces sensory nerve growth. Osteoblasts release PGE 2 to sensitize sensory nerves, transmitting ascending signals. In a weightless state, osteoblasts in the bone marrow exhibit reduced PGE 2 expression, leading to weakened ascending signals and increased sympathetic nerve tone. NE secreted by the sympathetic nerves binds to Adrb2 receptors on osteocytes, promoting high expression of RANKL, which activates osteoclasts and causes bone loss. From an energy perspective, decreased PGE 2 levels in bone lead to increased expression of NPY in the hypothalamus. NPY inhibits osteogenesis through two mechanisms: directly by suppressing the differentiation of osteoblast precursors into osteoblasts, and indirectly by inhibiting the production of free fatty acids (FFAs) in adipose tissue, which are essential for osteoblast differentiation and energy supply

The primary role of the skeleton is to provide mechanical support to the body,^152^ making mechanical stimulation the most frequent external input encountered by bone.^153,154^ It is an established fact that bone adapts to mechanical loading through a dynamic balance of resorption and formation.^155^ Surprisingly, mechanical signals have been found to participate in the regulation of bone by the brain through skeletal interoception (Fig. 3).^156^ Low back pain (LBP) is a hallmark symptom of spinal degeneration. Aberrant loading of the spine is a significant contributing factor to low back pain. Lumbar instability in mice serves as a classic model for studying LBP. In the study on the mechanisms underlying LBP, Cao group identified the porous endplate as a region of low bone density.^157^ Reduced bone density leads to increased relative mechanical load per unit bone area, promoting osteoblasts on the bone surface to secrete higher levels of PGE2. This process induces spinal sensitization, resulting in LBP. These findings further highlight the role of the PGE2/EP4 signaling pathway in skeletal interoception, where it mediates the detection of changes in bone density. Additionally, PGE2 emerges as a biochemical indicator of mechanical loading on bone.^35,37^ The imaging features of late-stage arthritis and spinal degeneration show substantial overlap, characterized by joint or intervertebral disc space narrowing, osteophyte formation, and subchondral bone alterations.^158^ This overlap is understandable, as both joints and the spine are major load-bearing structures in the body and are frequently subjected to shear forces, particularly in the case of joints.^159,160^ Notably, in advanced osteoarthritis (OA), aberrant ingrowth of type H vessels and sensory nerve innervation has been observed in the subchondral bone plate,^161^ a phenomenon similarly occurring in degenerated endplates.^157^ Furthermore, elevated levels of PGE2 in the subchondral bone have been prominently detected in DMM and ACLT OA mouse models.^36,162^ Another intriguing phenomenon is the bone loss observed in astronauts in space flight.^163,164^ While the confined environment of space may exert negative psychological effects that indirectly contribute to bone loss, microgravity is widely regarded as the primary factor directly driving the reduction in bone mass among astronauts.^165,166^ Guo and colleagues investigated the mechanisms of weightlessness-induced bone loss using a hindlimb unloading (HU) mouse model.^38^ Their study also highlighted the mediating role of PGE2 in the regulation of bone mass under gravitational influence. They observed a significant decrease in the levels of PGE2 in unloaded bone, which triggered a cascade of events leading to increased sympathetic nervous tone and enhanced secretion of NE. Sympathetic-derived NE binds to Adrb2 on osteocyte, thereby upregulating its expression of RANKL, which promotes osteoclast differentiation within the bone and results in bone loss.^38^ Notably, the study further revealed that the decline in PGE2 expression in unloaded bones significantly increased hypothalamic Neuropeptide Y (NPY) expression.^38^ NPY is a signal molecule that promotes energy storage.^167^ It enhances glucose uptake and conversion into triglycerides by adipocytes, potentially by influencing insulin sensitivity or other metabolic pathways, while simultaneously inhibiting fatty acid oxidation. This mechanism facilitates the retention of lipids within adipose tissue depots.^168–170^ Interestingly, the increase in hypothalamic NPY and sympathetic tone caused by the reduction of PGE2 exerts opposing effects on lipolysis (Fig. 3). This results in a paradoxical phenomenon where the adipose tissue weight in mice remains unchanged despite elevated levels of free fatty acids.^38,171^ Mechanistically, PGE2 continues to act as a trigger in the skeletal interoception system.^33^ Elevated PGE2 levels in the bone marrow activate the expression of the hypothalamic co-repressor SMILE via phosphorylated CREB (pCREB), leading to the downregulation of NPY expression in the arcuate nucleus (ARC).^172^ The ARC NPY signaling pathway inhibits sympathetic tone, which in turn downregulates the expression of uncoupling protein 1 (UCP1) in brown adipose tissue through tyrosine hydroxylase-containing neurons.^173,174^ This suppression of UCP1 expression attenuates lipolysis and thermogenesis in brown fat.

Whether in the context of skeletal interoception’s sensitivity to mechanical stimuli or its regulation of energy balance, it is evident that the PGE2/EP4 signaling pathway serves as a critical trigger in these processes.^34^ Bone reflects its physiological state through the level of PGE2, thereby initiating a cascade of subsequent reactions. Is PGE2/EP4 signaling the only pathway through which sensory neurons perceive bone homeostasis? Current research has not definitively identified other signaling pathways involved in skeletal interoception. However, for a signaling pathway to be considered part of the skeletal interoception system for sensing bone homeostasis, it must meet the following criteria: (1) Changes in the level of signaling molecules can serve as markers of changes in bone homeostasis. It means that it’s unnecessary to notice which happens before the other; (2) Signaling molecules can activate sensory neurons; (3) Sensory neuron stimulation activated by signaling molecules can be transmitted to the brain and affect the expression of genes or substances related to neural conduction in central neurons; (4) Central processing of the signals activated by signaling molecules will regulate bone homeostasis through descending pathways, such as the SNS, the parasympathetic nervous system, or other nerves. NGF derived from osteoblasts has the potential to mediate skeletal interoception. This is because it binds to the tropomyosin receptor kinase A (TrkA) receptor, a sensory receptor widely expressed by nerves distributed on the surface of adult bones,^175^ initiating various signaling pathways related to the survival, proliferation, and differentiation of neural cells.^176^ This meets the basic condition for bone-central interaction, which is a signaling pathway connecting bones and nerves. Tomlinson and colleagues demonstrated that the NGF/TrkA signaling pathway plays an important role in bone formation, which meets condition (1) mentioned above.^177^ A recent study by Cao also showed that the deletion of TrkA in sensory neurons reduced the formation of the talus and mechanical load-induced bone formation.^35^ Whether the NGF/TrkA signaling pathway can trigger a central response by activating sensory nerves and then assign an “executor” to regulate bone homeostasis remains a key question for this pathway to be considered another sensing route in skeletal interoception. Further research is needed to address this question.

Inflammation is a common phenomenon associated with intervertebral disc degeneration and arthritis.^158^ PGE2, as a critical trigger within the skeletal interoception system, is closely associated with inflammation. Microsomal prostaglandin E synthase-1 (mPGES-1) is a PGE synthase that couples with cyclooxygenase-2 (COX-2) and plays a pivotal role in the synthesis of PGE2 under pathological conditions such as inflammation.^178^ Brain inflammation similarly poses a threat to neuronal health, impairing the processing of afferent signals and the transmission of efferent signals.^179,180^ Therefore, alleviating inflammation may have a beneficial impact on the entire skeletal interoception system. It is a widely accepted fact that physical activity promotes overall health and well-being.^181^ Regular aerobic exercise has been shown to reduce levels of inflammatory markers such as C-reactive protein (CRP), interleukin-6 (IL-6), and tumor necrosis factor-alpha (TNF-〈), while promoting the production of anti-inflammatory factors such as interleukin-10 (IL-10), interleukin-1 receptor antagonist (IL-1RA), interleukin-4 (IL-4), and transforming growth factor-beta 1 (TGF-®1).^182,183^ For instance, regular aerobic exercise in patients with metabolic syndrome has been shown to decrease IL-6 levels by 30%, TNF-〈 by 15%, and white blood cell count by 15%.^184^ Furthermore, consistent physical activity also exerts anti-inflammatory effects on chronic and inflammatory diseases. Similarly, moderate-intensity aerobic exercise performed weekly can help reduce peripheral inflammation levels in patients with type 2 diabetes.^185^ Routine physical exercise enhances the chemotaxis of neutrophils and natural killer (NK) cells, optimizing their functional states.^186^ For example, after leg and chest strength training, elderly individuals with an average age of 71 exhibited a threefold reduction in the levels of pro-inflammatory monocytes (CD14 and CD16) in their blood.^187^ Additionally, regular physical exercise, including both aerobic and resistance training, has been shown to decrease the secretion of pro-inflammatory cytokines in young adults and stimulate the release of anti-inflammatory mediators such as IL-6 from skeletal muscles.^188^ Exercise-induced increases in circulating IL-6 levels correspond with elevated plasma concentrations of anti-inflammatory cytokines, including IL-1RA and IL-10. IL-1RA inhibits IL-1® signaling, while IL-10 suppresses the production of pro-inflammatory cytokines such as TNF-〈.^189^ Moreover, prolonged exercise also influences the number of different T cell populations, including regulatory T cells, which play a key role in immune system regulation.^190^ Thus, exercise may represent a crucial mechanism for modulating immune and inflammatory responses. Osteoclasts play a critical role in the functioning of skeletal interoception by resorbing calcified tissue, creating niches that facilitate the ingrowth of nerves and blood vessels. The anti-inflammatory effects of exercise effectively suppress osteoclast activity, thereby mitigating joint or spinal degeneration to some extent and alleviating the pain associated with these conditions.^191^

In conclusion, research on skeletal interoception has just begun. This is an intriguing field with many unknowns waiting to be uncovered. Are there more signaling pathways involved in skeletal interoception? Does the source of signaling molecules affect the activation of skeletal interoception? Are higher brain regions involved in processing incoming signals? What specific brain regions are involved in processing skeletal interoception signals? Are the executors of central commands only the autonomic nervous system? Can the brain directly regulate bone homeostasis? Moreover, skeletal interoception is not just a one-way regulation from the brain to the bones. From the perspective of bones, utilizing signals released by bones to achieve reverse regulation of the brain is also a promising research direction. Finally, treatment strategies based on the theory of skeletal interoception will be the practical application of this concept. It has been proven that PTH alleviates LBP by affecting sensory nerve innervation in porous endplates,^37^ and PTH secretion has also been shown to be regulated by the central nervous system.^192^ Such a central endocrine-bone treatment pathway holds promise for being integrated into the skeletal interoception system.

## Design of biomaterials for bone-brain interaction under materiobiology

In the previous sections, we have thoroughly examined the complex mechanisms of bone-brain interaction, particularly the bidirectional regulation mediated by biological signals such as cytokines and EVs. However, these regulatory processes are prone to disruption by diseases or injuries, and relying on neuronal damage in conditions like TBI to accelerate bone fracture healing is not a viable therapeutic strategy. Nevertheless, these mechanisms provide a biomimetic foundation for the development of biomaterials. Moreover, traditional drugs often suffer from limitations such as single-target specificity, systemic toxicity, and rigid modes of action, underscoring the need for biomaterials that can complement or even lead drug delivery by enhancing the physiological environment and mitigating adverse effects. This is well reflected in the evolution of bone biomaterials, where the focus has shifted from purely structural biomimicry to functional biomimicry in material design. In 2017, Liu proposed the concept of “materiobiology”, which is a scientific discipline that studies the biological effects of the properties of biomaterials on biological functions at the cell, tissue, organ, and whole organism levels.^23^ Materials biology focuses on how materials can induce tissue regeneration through their biological effects in vivo, rather than preparing complex functional tissue-like or organ-like devices in vitro.^23^ Designing materials that can interact with the implantation site and improve the local microenvironment to achieve tissue repair or ameliorate systemic aging aligns with the principles of materials biology. Previously, we discussed various signaling pathways and mediating molecules involved in bone-brain interactions, indicating the extensive connections between bone and brain. Research on bone-brain interactions is expected to address many currently incurable diseases, such as AD,^193^ stroke,^194^ and osteoporosis.^195^ However, independent protective mechanisms, such as the BBB, cerebrospinal fluid circulation, and a relatively independent immune system, have been established to protect the brain with its highly organized neuronal networks.^196,197^ Therefore, unlike biomaterials designed for isolated applications in either bone or brain, designing materials that mediate interactions between bone and brain must consider the pathways of these interactions. It is crucial to ensure that the intervention does not negatively impact the brain’s environment. The following section reviews materials that utilize the mechanisms of bone-brain cross-organ interactions and explores potential design strategies for such materials.

**Fig. 4:**
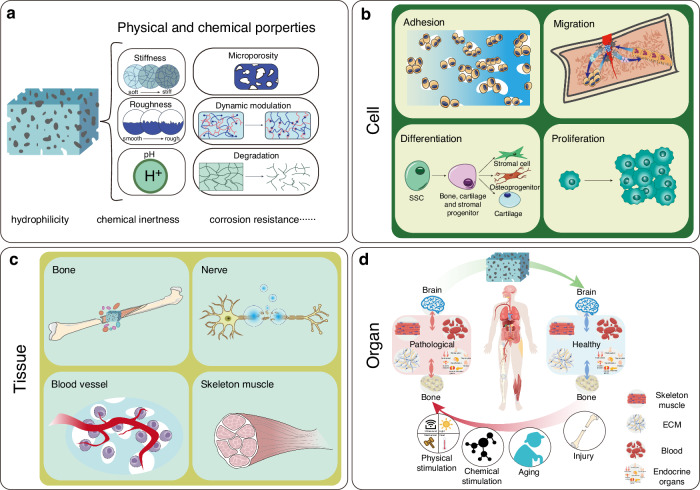
A schematic diagram showing the role of materiobiology in bone-brain interactions. **a** The physicochemical properties of materials with biological effects; **b** At the cellular level, materials provide attachment sites for osteoblasts, promote the migration of osteoblasts and osteoclasts, regulate the differentiation of skeletal stem cells into stromal cells, osteoprogenitors, and cartilage progenitors, and promote the proliferation of related cells; **c** At the tissue level, materials release bioactive factors that promote bone regeneration, nerve repair, Vascularization, and skeletal muscle development; **d** At the organ level, materials exert biological effects that counteract the negative cross-organ impacts on the bone-brain axis caused by physical stimulation, chemical stimulation, aging, injury, and other factors

The core concept of materiobiology lies in understanding and designing the interactions between materials and biological systems to achieve precise regulation of biological processes. By integrating principles from materials science and biology, materiobiology aims to develop functional materials that actively influence cellular behavior, signal transmission, tissue repair, and regeneration. These materials are not merely structural supports; they serve as active modulators capable of participating in and regulating the dynamic processes within biological systems (Fig. 4). Bone-brain interaction, as an inter-organ study, presents a promising material intervention point, with pathways of interaction primarily involving cytokines and EVs. Additionally, systemic inflammatory storms resulting from bone-brain homeostasis disruption are a critical aspect of their mutual influence.

Therefore, in the context of bone-brain interaction, material design guided by materiobiology should focus on creating smart materials that can precisely regulate the transport of cytokines or EVs, thereby enhancing signal transmission between bone and brain. By adjusting the chemical properties, surface functionalization, and mechanical characteristics of these materials, it is possible to optimize the release and absorption mechanisms of these signaling molecules. Additionally, these materials should be bioactive, capable of improving the microenvironment between bone and brain by releasing specific bioactive molecules or interacting with surrounding tissues to stabilize and optimize the bone-brain microenvironment. Moreover, the materials should be dynamically responsive, able to adjust to changes in the biological environment, such as fluctuations in pH, temperature, or biomolecule concentrations, thereby modulating key biological activities in real-time. Furthermore, the materials should facilitate bidirectional signal transmission, ensuring effective cellular communication between bone and brain while simultaneously supporting bone regeneration and neural repair, thus maintaining the overall health of the bone-brain system. Through these strategies, materiobiology offers innovative material solutions to address the complex challenges of bone-brain interactions, ultimately optimizing the regulation of the bone-brain microenvironment and improving therapeutic outcomes for related diseases.

Through these design strategies, materiobiology holds promise for providing innovative material solutions to address the complex challenges of bone-brain interactions, ultimately optimizing the regulation of the bone-brain microenvironment and improving therapeutic outcomes for related diseases.

### Simulation of bone-brain signaling mechanisms

As previously described, bone-/brain-derived factors and EVs are the primary means by which the bone-brain interaction occurs. Dysfunction of the bone and brain can lead to disruption of bone-brain signaling.^198^ Therefore, developing bioactive materials that simulate bone-brain signaling mechanisms represents a potential application of bone-brain interaction principles. EVs, a type of natural nanocarrier with excellent biocompatibility, have the capability to encapsulate and deliver bioactive molecules (proteins, lipids, nucleic acids). More importantly, they can effectively cross the BBB and deliver drugs to the brain.^199,200^ Studies have found that brain-derived EVs rich in osteogenic-related miRNAs can target bones following nerve injury,^104^ and that brain-derived EVs in AD can cross the BBB and reach distal bone tissue, inhibiting the osteogenic differentiation of BMSCs and promoting their adipogenic differentiation.^10^ Xie and colleagues found that exosomes from young osteocytes can be transported to the brain and play a protective role in the AD pathway, a function that is lost in exosomes from elderly osteocytes due to changes in their contents.^201^ This demonstrates that EVs are important tools in bone-brain signaling and that it is necessary to control the release of bone-derived factors in delivery systems to achieve better therapeutic effects. The application of natural EVs in drug delivery provides significant inspiration for the development of artificial biomaterials. The structural lipid membrane composition of EVs ensures the integrity of molecular cargo and facilitates transmembrane transport, providing a basis for the development of artificial liposomes. EVs participate in intercellular communication through receptors and ligands on their membrane surface.^202,203^ EVs utilize surface functional molecules to penetrate the BBB, a technique referred to as the “molecular Trojan horse,” which is used for surface modification of nanoparticles.^204^ Neutrophil-derived EVs contain functional molecules from the neutrophil membrane, enabling them to spontaneously migrate to inflammation sites.^205^ This feature is harnessed in inflammation-targeted technologies that coat nanoparticles with neutrophil membranes. Leveraging the natural structural advantages of EVs and their critical role in bone-brain interactions, we propose that designing nanoparticle carriers to mimic the structure of EVs represents an effective strategy for enriching and targeting bioactive molecules involved in bone-brain interactions, as well as the active components of natural EVs, thereby enabling controlled modulation of bone-brain dysregulation.

### Material design strategies for bone-brain interactions

The BBB is composed of brain endothelial cell, which work in conjunction with pericytes, smooth muscle cells, astrocytes, microglia, and macrophages to form the vascular wall. Together, they provide nutrients to the brain and isolate harmful substances.^206^ The BBB’s role in blocking substances is a crucial component of the brain’s protective mechanisms, which significantly limits drug delivery. Many studies aim to improve drug delivery efficiency by mildly disrupting the BBB. Optical/photothermal therapy, electrical stimulation, and acoustic/mechanical stimulation are three common physical methods used to modulate BBB permeability.^207^ However, these methods have various issues related to targeting specificity and safety. For instance, they compromise the integrity and physiological function of the BBB, which can lead to the accumulation of blood components, foreign substances, and exogenous drugs within the BBB, potentially causing damage to the brain.^208^ Even though focused ultrasound has become a leading clinical application technology that can non-invasively modulate the BBB locally with minimal damage to surrounding tissues, these uncertain and difficult-to-control damages pose significant safety risks for an organ as delicate and complex as the brain.^207^ Additionally, in the case of AD, BBB disruption leading to the entry of peripheral Aβ can exacerbate the progression of AD, and AD itself can cause BBB dysfunction.^209^ Therefore, non-disruptive delivery strategies would be a more feasible approach.

### Size and material

Constructing nanoparticles (NPs) is one of the most versatile and promising tools for drug delivery across the BBB. One of the prerequisites for NPs to penetrate the BBB is their sufficiently small size. The endothelial cells of the BBB have tight junctions that allow molecules with a molecular weight of less than 400 Da to pass through by passive diffusion.^204^ The optimal size for NPs is 10–100 nm, as NPs smaller than 5 nm are easily cleared by the kidneys.^210^ To penetrate the BBB, which is composed of highly lipophilic endothelial cells, NPs are typically designed as liposomes or polymer-based nanoparticles.^211^ The recent success of Moderna and Pfizer/BioNTech’s COVID-19 gene vaccines, which utilize liposome delivery systems, has brought significant attention to this category of nanoparticles.^212^ Similar liposome technology has also been used for brain delivery. For example, nucleic acid-based therapeutic drugs have been delivered to the brains of animals using surface-modified liposomes.^213^ Seju and colleagues developed PLGA nanoparticles with a particle size of 91.2 ± 5.2 nm loaded with olanzapine.^214^ In vivo data showed that this system, administered intranasally, achieved efficient brain delivery and reduced first-pass metabolism in the liver.^214^ Cao and colleagues developed a ROS-responsive nanocarrier, RAPA@tRPCS, for the targeted delivery of the neuroprotective agent rapamycin (RAPA) to alleviate ischemic brain injury.^215^ This nanocarrier consists of a sulfonated chitosan polymer core, modified with ROS-responsive boronate, and encapsulated in a red blood cell membrane shell containing a stroke-homing peptide. In vivo experiments on a mouse model of transient middle cerebral artery occlusion showed that the intravenously injected nanocarrier RAPA@tRPCS effectively polarized microglia, maintained BBB integrity, reduced cerebral infarction, and promoted neurovascular remodeling in the brain.^215^

### Targeted modification

Apart from implanting devices directly into lesions or injecting drugs directly into the lesion site, other delivery methods inevitably need to address targeting issues, especially for organs like bones that are distributed throughout the body (Table 2). For brain diseases, direct drug administration to the brain often causes fear and resistance in patients, and indeed poses greater risks. Designing biomaterials to target or mimic the microenvironment of specific cell populations can achieve precise regulation of biological processes.^216^ Therefore, targeted modification of drug-carrying materials is an essential aspect of developing bone-brain interaction materials.

Viral vector-based gene therapy has been widely studied and applied in clinical settings.^138^ For example, the FDA-approved Luxturna and Zolgensma AAV gene delivery systems have been successfully applied to the human central nervous system, treating Leber’s congenital amaurosis and spinal muscular atrophy.^217^ However, the inability of viruses to specifically target microglia and macrophages limits the application of viral vectors in treating microglia-related diseases.^138^ Non-viral nanoparticle carriers stand out due to their flexible modifiability. The targeted modification of non-viral nanoparticles can be categorized into modifications of the physicochemical properties of the nanoparticle surface and the attachment of functional groups. Surface charge is a critical physical parameter for penetrating the BBB.^218^ In studies involving eight human cell lines, it was found that positively charged nanoparticles have a higher cellular uptake rate compared to negatively charged and neutral nanoparticles.^219^ This is due to the presence of a large amount of negatively charged proteoglycans in the endothelial cells of the BBB.^220^ Surface modification with various functional molecules is also an important approach to enhance the targeting capability of nanoparticles. Through chemical modifications, specific ligands or antibodies can be attached to the surface of nanoparticles to recognize and bind to target cells.^221^ For example, polyethylene glycol modification can increase the biocompatibility and circulation time of nanoparticles, reducing recognition and clearance by the immune system.^222^ This type of modification not only improves the stability of nanoparticles but also allows for precise delivery to specific cells or tissues by attaching targeting ligands. Peptides are also a common means of surface modification for NPs. For example, the SDSSD peptide can specifically recognize and bind to osteoblasts. By modifying polyamidoamine dendrimers with this peptide, a multifunctional RNA delivery system is formed, significantly enhancing drug accumulation and therapeutic effects in bone tissue.^223^ Stroke-homing peptide modification is another mature technique that precisely targets stroke sites by specifically binding to apoptotic neurons in the affected area.^224^ Peptide-modified nanoparticles have shown great potential in improving drug targeting, overcoming biological barriers, promoting personalized medicine, and expanding application fields. With continuous advancements in nanotechnology and biotechnology, the design and application of peptide-modified nanoparticles will become more precise and efficient, providing new solutions for the treatment of various diseases.

### Cell membrane coating technology

Cell membrane coating technology is an emerging method for nanoparticle surface modification, commonly using red blood cell membranes and neutrophil membranes, among others. By employing this technique, nanoparticles can mimic the intrinsic biological functions of different cell membranes from biological systems, thereby enhancing their targeting capability, retention, and biocompatibility.^225,226^ This surface modification method has been shown to have potential and significance for biomedical applications.

Red blood cells (RBCs) are the most abundant type of blood cell in human blood. The enucleated structure of mature RBCs and their ease of isolation make them an ideal source for cell membranes. The main advantages of RBC membrane coating technology include superior biocompatibility,^227^ limited immunogenicity,^228^ flexibility, and prolonged circulation time.^229^ Red blood cell membrane coating technology has been extensively studied in tumor therapy.^230^ Su and colleagues enhanced the stability of mesoporous silica nanoparticles (MSNs) by coating them with red blood cell membranes.^231^ Compared to uncoated MSNs, RBC membrane-coated MSNs were less prone to aggregation in physiological saline and other buffer solutions, maintaining a stable suspension. Due to the natural evasion capabilities of RBC membranes, RBC-coated MSNs could avoid recognition and clearance by the body’s reticuloendothelial system. This prolonged the presence of the drug in the bloodstream, aiding in the delivery of the drug to tumor sites. However, the RBC membrane coating method lacks active and specific targeting functions.^232,233^ While the mentioned RBC membrane coating can extend the circulation time of NPs in the bloodstream, which indeed helps increase the likelihood of the drug reaching the tumor, this passive targeting approach is not efficient enough. Fortunately, RBC membranes have good modifiability, and through some functionalization measures, precise targeting of RBC membrane-coated NPs can still be achieved. Zhang and colleagues used a lipid insertion method to modify RBCM-NPs, adding recombinant anti-EGFR-iRGD to the particle surface.^227^ This allowed the NPs to successfully and accurately target tumors in a high EGFR-expressing colorectal cancer model, whereas unmodified NPs showed poor efficacy.

In contrast, neutrophils have an innate ability to target inflammation because they highly express CXCR2 and lowly express CXCR4 in the blood.^234^ This expression pattern facilitates their infiltration into sites of inflammation.^235^ Therefore, neutrophil membranes are used to coat numerous anti-inflammatory drug delivery systems, eliminating the need for artificial targeting modifications and simplifying the material preparation process. As the most common type of white blood cell in the human body, neutrophils ensure the source and biocompatibility of the membrane. In the context of stroke, neuroinflammation is a major cause of ischemia/reperfusion injury.^236^ Zhao developed neutrophil membrane-coated polyprodrug nanoparticles (NRNs) loaded with fingolimod hydrochloride (FTY720).^237^ To characterize the targeting ability of NRNs, they intravenously injected Cy7-labeled NRNs and compared them to non-neutrophil-coated RNs in a control group. The results showed that in the RN group, nearly identical fluorescence signals were detected in both hemispheres of the stroke mice’s brains. In contrast, in the NRNs group, the fluorescence intensity in the inflammation site of MCAO mice was 4.43 times higher than in the contralateral brain, indicating that the neutrophil membrane-modified drug delivery system had stronger selective distribution and inflammation-targeting capabilities. Additionally, the fluorescence intensity in the brains of the NRNs group was approximately 15.2 times higher than that of the RNs group, demonstrating the BBB penetration and brain retention capabilities conferred by neutrophil membrane modification. Additionally, studies have found that neutrophil expression of CXCR2 and CXCR4 can change, resulting in dual targeting capabilities. In aging, neutrophils exhibit high expression of CXCR4 and low expression of CXCR2, shifting their targeting from inflammation sites to the bone marrow.^238^ You utilized this characteristic to deliver drug-loaded poly(lactic-co-glycolic acid) nanoparticles by hitchhiking on neutrophils, crossing the bone marrow-blood barrier to target the bone marrow.^239^ Within two hours, a significant and increasing number of FDG-labeled neutrophils were detected in the bone marrow. The superior targeting capability also reduced the likelihood of the drug delivery system being absorbed by the liver and other metabolic organs or high-energy-demand areas such as muscles and the brain, thereby reducing the toxic side effects of some drugs.

### Designing materials for bone-brain interactions with the aid of AI

Biomaterials hold great potential for enhancing drug delivery and promoting effective signaling pathways between bone and brain through optimized microenvironments.^240^ However, designing these materials is complex, as it requires balancing multiple factors such as the base material’s physicochemical properties, biocompatibility, and bioactive elements’ roles in tissue repair.^241^ This complexity makes traditional methods insufficient for identifying optimal material combinations efficiently. In this context, AI’s capabilities in high-throughput computation have become crucial.^242^ AI not only accelerates the screening of material systems for desired biological effects but also refines material and bioactive combinations to meet specific biological requirements, thus enhancing both the speed and accuracy of biomaterial development.^243,244^

AI, driven by rapid advancements in computing, has transformed from “Internet+” to “AI+” in just a decade, profoundly impacting scientific research and daily life.^245,246^ Companies like Google and Microsoft have launched AI models like GNoME and MatterGen for materials science, signaling AI’s vast potential.^247^ Previously, AI transformed biopharmaceuticals by enabling atomic-scale drug molecule screening, a critical tool for major pharmaceutical R&D pipelines.^248^ With its powerful data processing, AI reduces trial-and-error costs and boosts efficiency, making it indispensable for biomaterials design.

**Fig. 5:**
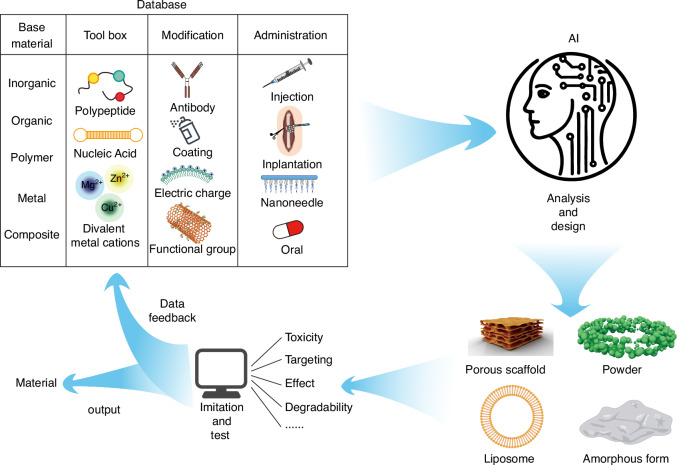
AI driven design of novel biomaterials and application in bone-brain interaction. A database containing information on the physicochemical and biological properties of materials, along with functional molecules, modification techniques, and administration methods, has been established. AI retrieves relevant data from the database based on specific requirements and, through high-throughput analysis and comparison, designs potential material solutions. These solutions are then simulated and tested for feasibility, with the simulation results fed back into the database to further refine and enhance its development

Under the concepts of “materiobiology” and “materials genomics,^249^ establishing a biomaterials database and leveraging AI technology could bring revolutionary changes to the design and development of biomaterials. The process involves initially obtaining materials science data through theoretical calculations, producing large volumes of such data via high-throughput computing, and then feeding the data into AI models.^250^ Finally, these AI models can infer the properties of unknown materials (Fig. 5).^251,252^ This outlines the technological roadmap for AI-driven transformation in materials design. Clearly, data is the foundation of this system.^253^ Despite advancements in materials genomics leading to the establishment of databases like the Materials Project,^254^ which includes biomaterials, there is still a lack of specialized databases for biomaterials. Therefore, to utilize AI in biomaterials design, the most crucial step is to establish a database that comprehensively interprets the biological properties of materials. Leveraging AI’s data integration capabilities can visualize the serial and parallel interactions in biological reactions, constructing a biological reaction network. This approach allows for the selection of appropriate materials to intervene at specific reaction nodes. Liu proposed the concept of a biomaterials “toolbox,” which elementizes biomaterials and emphasizes their functional characteristics.^255^ This concept can serve as the foundation for establishing a biomaterials database. Driven by specific needs, AI can select materials from the “toolbox” that meet these requirements and combine them. Then, through high-throughput simulations, AI can analyze a large number of combinations to find the optimal solution, achieving personalized customization.^256–258^ By quantitatively analyzing the synthesis, characterization, and other parameters involved at different stages, AI, with its unique modeling and predictive capabilities, plays a crucial role in design, synthesis, and characterization analysis.^259^ Additionally, clinical trials have always been the biggest hurdle for the actual implementation of biopharmaceuticals and biomedical materials due to limitations related to experimental subjects, safety, and ethical issues.^260–263^ AI can overcome these obstacles by using clinical data from already-approved materials to conduct simulations and virtual experiments, thereby improving the biocompatibility and efficacy of materials before clinical trials.^264^ Gu proposed three recommendations for the development of AI in the field of biomaterials: 1. Material Design Stage: Focus on establishing large-scale biomedical nanomaterial databases and descriptor models to enhance algorithm performance. 2. Material Synthesis Stage: Emphasize improving algorithm efficiency and diversity, leveraging AI to guide and support the material synthesis process. 3. Material Characterization and Analysis Stage: Design AI algorithms for comprehensive analysis of various characterization results and build visualization interfaces to assist researchers in deeply understanding experimental outcomes and improving data analysis efficiency.^265^

In summary, “AI+“ serves as an essential accelerator for the development of biomedical materials. When combined with bioinformatics technologies like multi-omics analysis, AI can uncover extensive interactions between bone and brain. With the assistance of AI, material-mediated bone-brain interactions are no longer isolated effects of a single molecule in a specific pathway, but rather the therapeutic outcomes of multifunctional materials exerting biologically controlled effects in a spatiotemporal manner.

## Conclusion and prospects

With the increasing recognition of bone as an endocrine organ, bone-brain interactions have emerged as a significant area of research, revealing the complex bidirectional communication mechanisms between the skeleton and the brain. Studies have shown that bone-derived factors play a critical role in maintaining bone homeostasis, while also influencing the nervous system, particularly brain function, highlighting the importance of bone-brain crosstalk. Moreover, the impact of BRDI on bone health further underscores the interdependent and reciprocal relationship between the two systems. The concept of skeletal interoception offers a novel perspective, revealing how bones interact with the nervous system to sense and regulate physiological states. This review emphasizes innovative approaches in biomaterial design inspired by bone-brain interaction mechanisms, aiming to optimize bone-brain communication through materiobiological effects, thus advancing new strategies for the treatment of neurodegenerative and skeletal diseases. Furthermore, the integration of AI provides unprecedented acceleration in both research and clinical translation, enabling faster development of novel biomaterials and the formulation of personalized therapeutic approaches, with considerable clinical potential. In conclusion, research on bone-brain interactions not only offers new directions for disease treatment but also provides profound insights into the complexity of biological systems.

Looking ahead, research on bone-brain interactions will continue to unfold across multiple layers and dimensions, exploring the intricate mechanisms at the molecular and cellular levels. With the aging population, the interplay between skeletal health and neural function is increasingly becoming a focus of medical research. This is particularly true in the treatment of osteoporosis, neurodegenerative diseases, and cerebrovascular disorders, where a deeper understanding of bone-brain interaction mechanisms will provide new breakthroughs in disease prevention and treatment. Future biomaterials will no longer be mere tools for treating fractures or skeletal diseases; they will evolve into multifunctional, intelligent systems capable of modulating bone-brain interactions. Through precise material design, these systems will facilitate the normal functioning and healthy balance between the skeleton and the brain. The introduction of AI and machine learning presents unprecedented opportunities in this field, enabling accelerated screening and optimization of new materials, while also offering powerful data support in the development of personalized treatment plans. Interdisciplinary collaboration will drive the deep integration of neuroscience, bone science, materials science, and AI, paving the way for more precise and effective therapeutic strategies. In summary, research into bone-brain interactions not only brings new hope for disease treatment but also provides an important strategic pathway for improving overall human health, with significant social value and practical implications, particularly in addressing the challenges of global aging and related diseases.
